# Supplementary material for: Modeling glioblastoma heterogeneity as a dynamic network of cell states
Source: Mol Syst Biol. 2021 Sep 16;17(9):e10105. doi: 10.15252/msb.202010105 (PMC8444284; doi:10.15252/msb.202010105)
Supplement: Supplementary file 6 — Source Data for Figure 5 [file MSB-17-e10105-s004.zip › Figure5A_sourcedata/GSEA_3017/nosigdb_stateA.GseaPreranked.1622795026485/LARSSON_STATE_6.html]

Details for gene set LARSSON\_STATE\_6[GSEA]

|  || Dataset | state53017 |
| Phenotype | NoPhenotypeAvailable |
| Upregulated in class | na\_neg |
| GeneSet | LARSSON\_STATE\_6 |
| Enrichment Score (ES) | -0.62034273 |
| Normalized Enrichment Score (NES) | -3.046462 |
| Nominal p-value | 0.0 |
| FDR q-value | 0.0 |
| FWER p-Value | 0.0 |
Table: GSEA Results Summary

  

Fig 1: Enrichment plot: LARSSON\_STATE\_6      
 Profile of the Running ES Score & Positions of GeneSet Members on the Rank Ordered List

  

| PROBE | GENE SYMBOL | GENE\_TITLE | RANK IN GENE LIST | RANK METRIC SCORE | RUNNING ES | CORE ENRICHMENT || 1 | FABP5 |  |  | 0 | 1.759 | 0.0678 | No |
| 2 | METRN |  |  | 186 | 0.387 | -0.1121 | No |
| 3 | HNRNPDL |  |  | 315 | 0.320 | -0.2345 | No |
| 4 | CITED1 |  |  | 377 | 0.301 | -0.2871 | No |
| 5 | SOX9 |  |  | 577 | 0.254 | -0.4868 | No |
| 6 | BRI3 |  |  | 684 | -0.294 | -0.5870 | No |
| 7 | S100B |  |  | 696 | -0.299 | -0.5871 | No |
| 8 | TSC22D4 |  |  | 716 | -0.312 | -0.5951 | No |
| 9 | ID2 |  |  | 727 | -0.323 | -0.5932 | No |
| 10 | HEY1 |  |  | 751 | -0.344 | -0.6041 | No |
| 11 | C1orf122 |  |  | 753 | -0.345 | -0.5919 | No |
| 12 | IRS2 |  |  | 781 | -0.368 | -0.6062 | Yes |
| 13 | PNRC1 |  |  | 786 | -0.371 | -0.5961 | Yes |
| 14 | TUBB2A |  |  | 792 | -0.375 | -0.5869 | Yes |
| 15 | BEX1 |  |  | 802 | -0.385 | -0.5816 | Yes |
| 16 | PHPT1 |  |  | 811 | -0.393 | -0.5748 | Yes |
| 17 | FHL1 |  |  | 814 | -0.397 | -0.5617 | Yes |
| 18 | HOPX |  |  | 821 | -0.403 | -0.5524 | Yes |
| 19 | FTL |  |  | 825 | -0.409 | -0.5399 | Yes |
| 20 | BNIP3L |  |  | 841 | -0.432 | -0.5390 | Yes |
| 21 | MAP1LC3A |  |  | 842 | -0.436 | -0.5222 | Yes |
| 22 | MT3 |  |  | 905 | -0.559 | -0.5659 | Yes |
| 23 | SAT1 |  |  | 909 | -0.573 | -0.5470 | Yes |
| 24 | FTH1 |  |  | 910 | -0.573 | -0.5249 | Yes |
| 25 | DKK3 |  |  | 912 | -0.579 | -0.5037 | Yes |
| 26 | RAB31 |  |  | 916 | -0.586 | -0.4843 | Yes |
| 27 | ZMAT3 |  |  | 929 | -0.650 | -0.4718 | Yes |
| 28 | ID1 |  |  | 931 | -0.651 | -0.4478 | Yes |
| 29 | CLU |  |  | 938 | -0.685 | -0.4277 | Yes |
| 30 | CEBPD |  |  | 939 | -0.690 | -0.4011 | Yes |
| 31 | CRYAB |  |  | 967 | -0.940 | -0.3933 | Yes |
| 32 | PBX1 |  |  | 968 | -0.964 | -0.3562 | Yes |
| 33 | LGALS3 |  |  | 970 | -0.989 | -0.3191 | Yes |
| 34 | FABP7 |  |  | 971 | -0.994 | -0.2809 | Yes |
| 35 | ID3 |  |  | 981 | -1.505 | -0.2324 | Yes |
| 36 | SPARCL1 |  |  | 984 | -1.810 | -0.1648 | Yes |
| 37 | NEAT1 |  |  | 986 | -2.049 | -0.0869 | Yes |
| 38 | GFAP |  |  | 987 | -2.255 | -0.0000 | Yes |
Table: GSEA details [plain text format]

  

Fig 2: LARSSON\_STATE\_6: Random ES distribution      
 Gene set null distribution of ES for **LARSSON\_STATE\_6**
